# Feasibility and usability of a wearable sensor-based telerehabilitation program for home-based exercise in patients with lower limb amputation: a pilot study

**DOI:** 10.3389/fresc.2026.1828171

**Published:** 2026-07-23

**Authors:** Ravi Shankar, Pei Shan Tong, Emily Yee, Poo Lee Ong

**Affiliations:** 1 Clinical Research & Innovation Office, Tan Tock Seng Hospital, National Healthcare Group, Singapore, Singapore; 2 Rehabilitation Research Institute of Singapore (RRIS), Lee Kong Chian, School of Medicine, Nanyang Technological University, Singapore, Singapore; 3 Department of Physiotherapy, Tan Tock Seng Hospital, Singapore, Singapore; 4 Institute of Rehabilitation Excellence (IREx), Tan Tock Seng Hospital Rehabilitation Centre, Singapore, Singapore; 5 Rehabilitation Medicine, Tan Tock Seng Hospital, Singapore, Singapore

**Keywords:** home-based exercise, inertial measurement unit, lower limb amputation, rehabilitation technology, telerehabilitation, wearable sensors

## Abstract

**Background:**

Lower limb amputation (LLA) presents significant rehabilitation challenges, with patients requiring comprehensive multidisciplinary care to achieve optimal functional outcomes. Telerehabilitation using wearable sensor technology offers a promising solution to improve therapy accessibility and exercise adherence among this population. This pilot study evaluated the feasibility, usability, and preliminary effects on hip strength, and user acceptability of a home-based telerehabilitation program using the Rebee wearable sensor system for patients with major lower extremity amputation.

**Methods:**

A prospective single-arm feasibility study was conducted at the Foot Care and Limb Design Centre, Tan Tock Seng Hospital, Singapore. Ten participants with unilateral major amputations (5 transtibial, 5 transfemoral) were enrolled. Participants performed daily home exercises using the Rebee inertial measurement unit (IMU) sensor system for 30 days, with remote monitoring and biweekly adjustments. Primary outcomes included hip extensor and abductor strength assessed using dynamometry at baseline (Week 0), Week 4, and Week 8. Secondary outcomes included quality of life (EQ-5D-5L), exercise compliance, patient satisfaction, and system usability (System Usability Scale).

**Results:**

All ten participants (80% male; median age 51.5 years, IQR 11) completed the intervention. Median hip extensor strength improved significantly from 8.43 kg at baseline to 9.87 kg at Week 4 (*p* < 0.05) and 10.88 kg at Week 8 (*p* < 0.05). Median hip abductor strength increased from 8.01 to 9.45 kg (Week 4) and 9.70 kg (Week 8), with statistically significant improvements (*p* < 0.05). Mean exercise compliance was 68%. The mean System Usability Scale score was 75.8, indicating favorable usability. Health-related quality of life showed mixed patterns: median self-rated health (EQ-VAS) increased from 80 at baseline to 87.5 by Week 8, whereas the proportion of participants reporting problems in several EQ-5D-5L dimensions did not improve and slightly increased. No adverse events were reported.

**Conclusions:**

Home-based telerehabilitation using the Rebee wearable sensor system is feasible, safe, and acceptable for patients with lower limb amputation, with preliminary improvements in hip strength. High user satisfaction and favorable usability support its potential. However, as a single-arm pilot without a control group, findings remain preliminary and not generalizable. Future RCTs are needed to confirm effectiveness and cost-effectiveness for clinical integration.

## 1 Introduction

Lower limb amputation (LLA) represents a significant global health challenge, with substantial implications for patients' physical function, mobility, and quality of life. Singapore has one of the highest rates of lower extremity amputations globally, with hospitals performing close to four amputations per day, primarily attributed to vascular complications from diabetes mellitus and peripheral arterial disease (1). While amputation surgery is often lifesaving, it generates considerable morbidity requiring comprehensive rehabilitation to mitigate impairments and optimize functional outcomes.

Rehabilitation following LLA is complex and multifaceted, requiring multidisciplinary team involvement to ensure optimal treatment outcomes and social integration (2). Physical activity participation after LLA is complicated by chronic conditions, severe disability, and unaddressed psychosocial factors, often resulting in prolonged rehabilitation processes that may extend to twelve months to achieve ambulation milestones with a prosthesis (3). Evidence supports that one to three exercise sessions of 20–60 min per week significantly improves balance, walking speed, walking endurance, and transfer ability in adults with LLA, particularly when combining aerobic exercises with lower limb strengthening (4).

Telerehabilitation has emerged as a valuable healthcare delivery modality for individuals with physical limitations who cannot regularly attend outpatient physiotherapy (5). Recent systematic reviews have demonstrated that telerehabilitation augmented by wearable sensor technology and artificial intelligence-driven feedback offers a scalable alternative to conventional physiotherapy, particularly valuable in addressing barriers including accessibility, adherence, and geographic constraints (6). Wearable inertial measurement units (IMUs) have shown particular promise in rehabilitation applications, enabling objective assessment of movement quality, exercise compliance, and functional outcomes (7). Recent systematic reviews of lower limb telerehabilitation support the utility of wearable technology for exercise assessment, joint angle estimation, and activity recognition (6), and unsupervised IMU-based monitoring of home exercise programmes has demonstrated feasibility for objectively quantifying adherence and movement quality outside the clinic (7).

For the amputee population specifically, wearable sensors can quantify mobility and assess gait characteristics during daily life, providing valuable objective data for prosthetic prescription and rehabilitation planning (8). The integration of sensor-based monitoring with mobile health platforms enables remote supervision, allowing patients to perform guided self-exercise programmes at home. This approach increases therapy intensity beyond what scheduled clinic sessions alone can provide, while reducing the burden of frequent hospital visits for patients and caregivers.

Despite growing evidence for telerehabilitation efficacy across various populations, limited research exists specifically examining wearable sensor-based home exercise interventions for patients with LLA. This population presents unique challenges including comorbidities, mobility limitations, and specific rehabilitation goals related to prosthetic preparation and use. Therefore, this pilot study aimed to evaluate the feasibility, usability, and preliminary effects of a home-based telerehabilitation program in patients with major lower extremity amputation, using the Rebee wearable sensor system, an IMU sensor paired with a tablet application that guides patients through exercises using an animated avatar (9).

The specific objectives were to: (1) assess the feasibility and safety of implementing a telerehabilitation program using wearable sensors in the community setting; (2) explore preliminary within-group changes in hip strength outcomes; and (3) determine patient satisfaction, exercise compliance, and system usability.

## 2 Methods

### 2.1 Study design

This was a prospective, single-arm pilot study conducted at the Foot Care and Limb Design Centre (FLC), Tan Tock Seng Hospital, Singapore. The study was approved by the National Healthcare Group Domain Specific Review Board (DSRB Reference: 2023/00126) and registered under the Human Biomedical Research Act (HBRA) framework. All participants provided written informed consent prior to enrollment. The study was conducted in accordance with the Declaration of Helsinki principles.

### 2.2 Participants

Inclusion criteria comprised adults aged 21–80 years with unilateral transtibial or transfemoral amputation attending the FLC or Centre for Advanced Rehabilitation Therapeutics (CART) clinic for amputee rehabilitation. Participants were required to follow two-step verbal commands consistently, have the ability to understand and provide consent, and perform self-exercise independently or with minimal assistance from one caregiver. Exclusion criteria included unstable cardiac or medical conditions, severe pain limiting movement or participation, severe cognitive impairment, requirement for two-person assistance for exercise or high fall risk without a competent caregiver, severe visual impairment, pregnancy, bilateral lower limb amputation, upper limb amputation, or inability to safely use the Rebee device as assessed during onboarding.

### 2.3 Intervention

#### 2.3.1 The Rebee system

**Figure 1:**
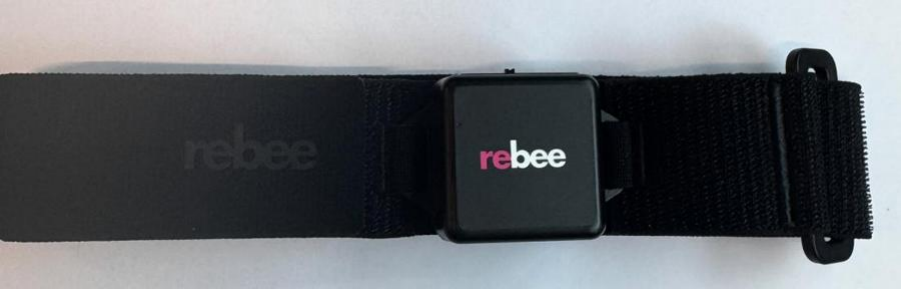
The Rebee (XCLR8) inertial measurement unit shown with its adjustable elastic strap. The sensor module measures 36 mm × 51.3 mm × 15 mm and weighs 13 g.

The Rebee wearable sensor (XCLR8, Singapore) is a commercially available IMU device validated for assessment of joint range of motion against optical motion capture systems (9). The system comprises a lightweight, compact sensor worn on the exercising limb that captures real-time movement data including joint angles, exercise repetitions, duration, and range of motion. The sensor incorporates a nine-axis IMU (tri-axial accelerometer, gyroscope, and magnetometer) and derives joint angles through sensor-fusion of these signals, sampling movement at a fixed rate of 20 Hz, the sampling frequency reported for this device in its validation study (9) and selected by the manufacturer to stream kinematic data to the paired application without perceptible latency in the real-time avatar; the full technical specifications and the device's validation against optical motion capture have been reported previously (9). The accelerometer, gyroscope, and magnetometer operated at manufacturer-specified full-scale measurement ranges of ±16 g, ±2,000°/s, and ±2 Gauss (±200 µT), respectively. The compact sensor unit measures 36 mm × 51.3 mm × 15 mm and weighs 13 g; a photograph of the device is shown in Figure 1. Prior to each exercise session, the sensor required a brief calibration step, during which it was held stationary in a defined neutral position to establish a reference orientation for subsequent joint-angle and repetition computation. Data is transmitted wirelessly to a mobile application on an Android tablet, which provides visual guidance, gamified feedback, and exercise tracking. A cloud-based dashboard enables remote monitoring of exercise performance and compliance by physiotherapists.

#### 2.3.2 Exercise protocol

Participants received one to two onboarding sessions at FLC or CART clinic, during which trained physiotherapists assessed their ability to safely perform prescribed exercises using the device. Caregivers were also educated on exercise supervision and emergency handling. A standardized home-based exercise program was prescribed, focusing on hip and knee strengthening and stretching exercises specifically designed for amputee patients. The exercise protocol was developed by the study physiotherapists, drawing on established principles of post-amputation strengthening and the individualized rehabilitation approach recommended in clinical practice guidelines (2), rather than adopted verbatim from a single published protocol. Exercises included: single leg bridging, modified bridging, inner range quadriceps, supine hip abduction, side-lying hip abduction, side-lying hip extension, seated knee extension, straight leg raise, supine hamstring stretch, and hip flexor stretch. These comprised supine, side-lying, seated, and bridging movements targeting the hip abductors, extensors, and flexors together with the knee extensors and hamstrings, and were demonstrated to each participant by the animated avatar within the tablet application. For each participant, three to seven exercises were selected from this menu by the supervising physiotherapist according to the individual's amputation level, baseline strength and range of motion, residual-limb and prosthetic status, comorbidities, fall risk, and ability to perform the movement safely. Not all ten exercises were appropriate or tolerable for every participant; the prescription was therefore tailored to target the most relevant impairments while avoiding contraindicated or unsafe movements, with a recommended total exercise duration of up to 60 min per day that could be completed in split sessions.

Participants performed home exercises using the Rebee sensor for 30 days, with remote (asynchronous) monitoring by the physiotherapist at weekly intervals through the cloud dashboard, which displayed sensor-recorded data on exercise repetitions, range of motion, session duration, and adherence rather than live video supervision. Exercise prescriptions were reviewed and adjusted approximately every 2 weeks based on these performance data; progression consisted of increasing the number of repetitions or sets, advancing to more demanding exercise variations, or substituting exercises as range of motion, strength, and tolerance improved, while reducing or modifying exercises that the participant found difficult or uncomfortable. Participants continued to receive standard care physiotherapy sessions once every 2 weeks throughout the study period.

### 2.4 Outcome measures

#### 2.4.1 Primary outcomes

Hip extensor and hip abductor muscle strength were measured using hand-held dynamometry at three time points: baseline (Week 0), Week 4, and Week 8 post-intervention. A hand-held dynamometer (Lafayette Manual Muscle Testing System, Model 01165; Lafayette Instrument Company, Lafayette, Indiana, USA; full-scale measurement range 0–1,334 N, equivalent to approximately 0–136 kg) was used, applied with a make-test technique; hand-held dynamometry has been reported to have good-to-excellent reliability and concurrent validity against fixed dynamometry for proximal lower-limb muscle groups (10). Hip extensors were tested in prone and hip abductors in side-lying, with the dynamometer placed at standardized landmarks and the joint in a defined neutral position. The hip extensors and abductors were selected because they are the principal muscle groups governing pelvic stability, stance-phase control, and balance during prosthetic ambulation and are therefore most relevant to fall prevention in this population; in lower-limb prosthesis users, hip abductor strength has been shown to relate to quiet-stance balance and gait performance, and the relevance of hip extensor and abductor strength to walking and balance has been demonstrated to differ by amputation level (11, 12); hip flexors and adductors, which contribute less directly to these functional goals and are more difficult to isolate reliably with hand-held dynamometry in patients with a residual limb, were not assessed. Three trials were performed for each muscle group, and the mean value was calculated. Measurements were conducted by trained physiotherapists using standardized positioning protocols (10).

#### 2.4.2 Secondary outcomes

Health-related quality of life was assessed using the EQ-5D-5L instrument, which includes five dimensions (mobility, self-care, usual activities, pain/discomfort, anxiety/depression) and a visual analogue scale (EQ-VAS) for overall health status. The EQ-5D-5L has demonstrated validity and reliability for use in the LLA population (13). Single-limb balance was assessed as the duration (in seconds) of unsupported single-leg stance on the intact limb, recorded at each time point where it could be performed safely. Because complete balance data could not be obtained for all participants across all time points, owing to safety considerations and feasibility, these data were not formally analyzed and are not reported. Exercise compliance was calculated as the percentage of days over the 30-day intervention period in which the participant completed at least two-thirds of their prescribed exercises, as monitored via the Rebee cloud dashboard. Patient satisfaction was evaluated using a 6-item Patient Satisfaction Scale, an investigator-developed instrument designed for this study (no previously validated scale specific to this context was available), with items rated on a 5-point Likert scale (1 = Strongly Disagree to 5 = Strongly Agree). System usability was assessed using the System Usability Scale (SUS), a validated 10-item questionnaire producing scores from 0 to 100, with scores above 68 indicating above-average usability (14, 15).

### 2.5 Statistical analysis

Given the pilot nature and small sample size, descriptive statistics were used to summarize participant characteristics and outcomes. Baseline comorbidity burden was characterized using the Charlson Comorbidity Index, a weighted index that assigns scores to a range of comorbid conditions, in which higher totals indicate a greater comorbidity burden and higher predicted mortality risk. Continuous variables were presented as median and interquartile range (IQR) due to non-normal distributions. The distribution of continuous variables was examined using the Shapiro–Wilk test; given the small sample and the non-normal distributions observed, non-parametric methods were used for inferential analysis. Categorical variables were reported as frequencies and percentages. Changes in hip strength across the three time points were analyzed using the Friedman test for repeated measures. *Post-hoc* pairwise comparisons were conducted using the Wilcoxon signed-rank test with Bonferroni correction for multiple comparisons. A one-tailed test was used to detect improvements in strength. Owing to the small sample and the ordinal, categorical nature of the individual EQ-5D-5L dimension responses, EQ-5D-5L outcomes were summarized descriptively (the number of participants reporting problems in each dimension and the median EQ-VAS) and were not subjected to inferential hypothesis testing, in order to avoid over-interpreting changes in a small sample; the Friedman and Wilcoxon tests were therefore applied only to the continuous hip-strength measures. Statistical significance was set at *p* < 0.05. Correlation between exercise compliance and strength changes was examined using Pearson correlation coefficient. All analyses were performed using SPSS version 26.0 (IBM Corporation, Armonk, NY, USA).

## 3 Results

### 3.1 Participant characteristics

Ten participants were enrolled and completed the study protocol. Table 1 presents the baseline demographic and clinical characteristics. The cohort comprised predominantly males (80%, *n* = 8) with a median age of 51.5 years (IQR 11). The ethnic distribution included Chinese (50%, *n* = 5), Indian (10%, *n* = 1), and Malay (40%, *n* = 4) participants. Five participants (50%) had transtibial (below-knee) amputations, while five (50%) had transfemoral (above-knee) amputations. The primary causes of amputation were vascular disease (70%, *n* = 7), including diabetes-related complications, trauma (20%, *n* = 2), and necrotizing fasciitis (10%, *n* = 1). The median Charlson Comorbidity Index score was 2 (IQR 0.75), indicating moderate comorbidity burden.

**Table 1:** Baseline demographic and clinical characteristics (*n* = 10).

Characteristic	Value
Age, years, median (IQR)	51.5 (46.5–57.5)
Gender, *n* (%)	
Male	8 (80%)
Female	2 (20%)
Ethnicity, *n* (%)	
Chinese	5 (50%)
Malay	4 (40%)
Indian	1 (10%)
Amputation level, *n* (%)	
Transtibial (BKA)	5 (50%)
Transfemoral (AKA)	5 (50%)
Cause of amputation, *n* (%)	
Vascular/diabetes	7 (70%)
Necrotizing fasciitis	1 (10%)
Trauma	2 (20%)
Osteomyelitis	0 (0%)
Charlson comorbidity index, median (IQR)	2 (2–2.75)

IQR, interquartile range; BKA, below-knee amputation; AKA, above-knee amputation.

### 3.2 Hip strength outcomes

**Figure 2:**
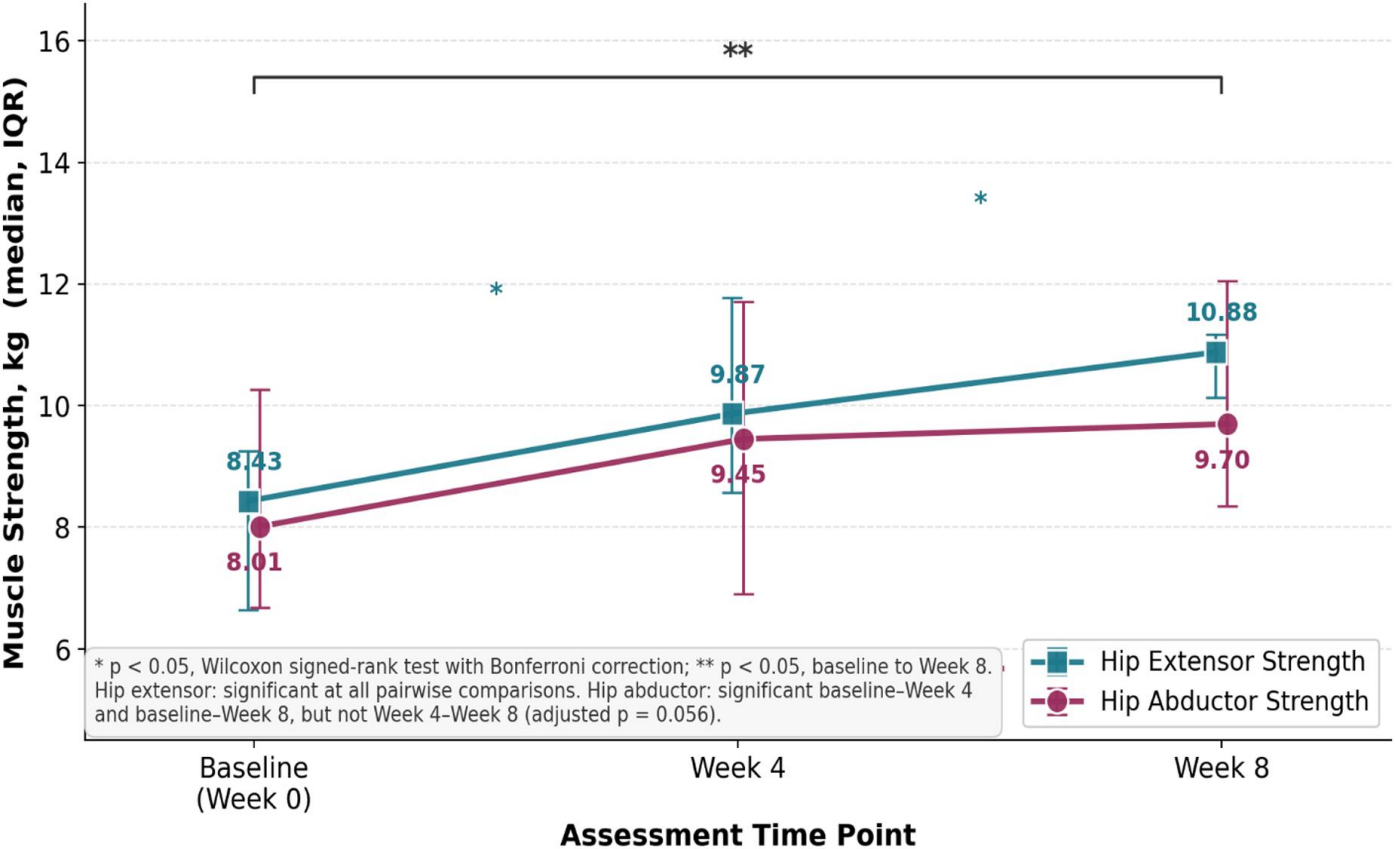
Line graph showing median hip extensor and hip abductor strength changes across Week 0, Week 4, and Week 8 time points.

Hip strength measurements demonstrated significant improvements over the 8-week study period (Table 2). Baseline median hip extensor strength was 8.43 kg (IQR 2.61). Participants showed progressive improvement to 9.87 kg (IQR 3.20) at Week 4 and 10.88 kg (IQR 1.05) at Week 8, representing an overall median improvement of 2.45 kg from baseline. These changes are illustrated in Figure 2. The Friedman test revealed statistically significant differences across time points for hip extensor strength (*p* < 0.05). *Post-hoc* pairwise comparisons using Wilcoxon signed-rank tests with Bonferroni correction confirmed significant improvements between baseline and Week 4 (*p* < 0.05), Week 4 and Week 8 (*p* < 0.05), and baseline and Week 8 (*p* < 0.05).

**Table 2:** Hip strength outcomes across time points (*n* = 10).

Outcome measure	Baseline (Week 0)	Week 4	Week 8	*p*-value*
Hip extensor strength (kg)	8.43 (2.61)	9.87 (IQR 3.20)	10.88 (IQR 1.05)	<0.05
Hip abductor strength (kg)	8.01 (3.59)	9.45 (IQR 4.80)	9.70 (IQR 3.70)	<0.05

Values presented as median (IQR).

* Friedman test for repeated measures.

Similarly, hip abductor strength improved from a baseline median of 8.01 kg (IQR 3.59) to 9.45 kg (IQR 4.80) at Week 4 and 9.70 kg (IQR 3.70) at Week 8, with a median improvement of 1.69 kg from baseline. The Friedman test confirmed statistically significant changes over time (*p* < 0.05). *Post-hoc* pairwise comparisons demonstrated significant improvements between baseline and Week 4 (adjusted *p* < 0.05) and between baseline and Week 8 (adjusted *p* < 0.05); the difference between Week 4 and Week 8 did not reach statistical significance (adjusted *p* = 0.056). The 95% confidence interval for hip extensor strength change was [2.23, 6.58], and for hip abductor strength was [1.14, 3.73], both indicating statistically significant improvements.

### 3.3 Exercise compliance and correlation analysis

Exercise compliance was defined as the percentage of days over the 30-day period in which the participant performed at least two-thirds of their prescribed exercises. Mean compliance was 68% (range 20%–100%). Individual compliance rates varied considerably, with individual rates ranging from 100% (full adherence) to 20% (lowest engagement). Participants completed a median of 22.5 ± 13 exercise days over the 30-day period. The mean compliance (68%) being lower than the median (75%, corresponding to 22.5 of 30 days) indicates a left-skewed distribution, in which most participants maintained relatively high adherence while a minority with low engagement reduced the overall mean. Correlation analysis between exercise compliance and strength changes yielded weak and non-significant associations. For hip extensor strength change, the correlation coefficient (*r*) was 0.3042 (*p* = 0.3928, 95% CI [−0.40, 0.78]). For hip abductor strength change, *r* = 0.3878 (*p* = 0.2681, 95% CI [−0.32, 0.82]). The confidence intervals including zero reinforce the lack of significant correlation between compliance rates and strength improvements in this small sample.

### 3.4 Quality of life outcomes

Health-related quality of life assessed via EQ-5D-5L showed mixed and divergent patterns across the study period (Table 3). At baseline, 70% of participants reported mobility problems (levels 2–5) and 70% reported problems with usual activities. By Week 8, the number of participants reporting problems did not decrease and in fact increased slightly in several dimensions, with mobility rising from 7 (70%) to 9 (90%), self-care from 3 (30%) to 4 (40%), and anxiety/depression from 2 (20%) to 3 (30%), while usual activities and pain/discomfort were essentially unchanged. In contrast, the median EQ-VAS, a single global self-rating of health, increased from 80 at baseline to 85 at Week 4 and 87.5 at Week 8. These two sets of findings are therefore discordant: the global EQ-VAS suggested better perceived health, whereas the dimension-level data did not show improvement and slightly worsened. Because these descriptive changes were not subjected to inferential testing and involve very small numbers, they should be interpreted with caution. The divergence may reflect small-sample fluctuation, the ordinal nature of the dimension levels, progression of underlying comorbidities or prosthetic transition over the study period, or increased awareness of difficulties as activity increased, rather than a genuine deterioration. Accordingly, we do not interpret the EQ-5D-5L data as evidence of quality-of-life improvement.

**Table 3:** EQ-5D-5L outcomes across time points.

EQ-5D-5L dimension	Baseline (*n* reporting problems)	Week 4	Week 8
Mobility (levels 2–5)	7 (70%)	7 (70%)	9 (90%)
Self-care (levels 2–5)	3 (30%)	2 (20%)	4 (40%)
Usual activities (levels 2–5)	7 (70%)	6 (60%)	7 (70%)
Pain/discomfort (levels 2–5)	4 (40%)	4 (40%)	4 (40%)
Anxiety/depression (levels 2–5)	2 (20%)	2 (20%)	3 (30%)
EQ-VAS, median	80	85	87.5

EQ-VAS, EuroQol visual analogue scale (0–100, higher scores indicate better perceived health).

### 3.5 Patient satisfaction and system usability

Patient satisfaction ratings were favorable across all evaluated domains (Table 4). The Patient Satisfaction Scale was completed by all 10 participants. All ten respondents (100%) agreed or strongly agreed that the system was easy to learn. Similarly, 100% found the setup comfortable, the training easy to complete at home, and the training not boring, and all respondents endorsed that the training was useful for exercising their legs and that home exercise programs should be part of standard therapy. The overall satisfaction scale indicated that 70% were extremely satisfied and 30% were somewhat satisfied with the system.

**Table 4:** Patient satisfaction scale results (*n* = 10 respondents).

Item	Agree/Strongly agree, *n* (%)
It is easy to learn how to use the system	10 (100%)
The setup was comfortable	10 (100%)
The training was easy to complete at home	10 (100%)
The training was not boring	10 (100%)
The training was useful for exercising my legs	10 (100%)
The home exercise program should be part of standard therapy	10 (100%)

The mean System Usability Scale (SUS) score was 75.8 (range 60–90), indicating favorable usability exceeding the benchmark of 68 for acceptable usability (15, 16). Nine participants (90%) scored above 70, suggesting high acceptability, while one participant scored below this threshold, indicating areas for improvement. According to established SUS interpretation frameworks, the mean score corresponds to an adjective rating of “good” usability.

### 3.6 Safety and technical issues

No adverse events, including falls or injuries, were reported during the study period. The Rebee sensor system was found to be safe for use, did not cause skin irritation, and did not interfere with prosthetic use in participants who received their prosthesis during the study. Technical challenges reported by participants included occasional connectivity issues, as the system requires both WiFi internet access and Bluetooth pairing between the tablet and sensor to function, tablet auto-shutdown during sessions, and sensor sensitivity calibration. Some participants with poor vision or lower technological familiarity required additional support during onboarding. Qualitative feedback suggested integrating a battery level indicator into the mobile application to facilitate recharge planning. Participants who were older or less technologically familiar experienced particular difficulty troubleshooting connectivity disruptions, leading to frustration and, in some cases, reduced exercise compliance.

## 4 Discussion

This pilot study suggests that home-based telerehabilitation using the Rebee wearable sensor system is feasible, safe, and acceptable for patients with lower limb amputation, and was associated with preliminary within-group improvements in hip strength. All enrolled participants completed the intervention, and statistically significant within-group increases in both hip extensor and abductor strength were observed over the 8-week study period. However, in the absence of a control group these changes cannot be attributed to the intervention. The favorable system usability scores and high patient satisfaction support the acceptability of this technology-enabled rehabilitation approach for this population.

Direct comparison of our strength findings with prior telerehabilitation trials in this population is limited, because the most directly comparable randomized controlled trial assessed different outcomes. In that trial of telerehabilitation-based structured exercise in individuals with unilateral transtibial amputation (two groups of 24 participants), the combination of telerehabilitation with home exercise produced significantly greater improvements than home exercise alone in functional and quality-of-life measures, including the Timed Up and Go test, the 30-second chair-stand test, the Activities-specific Balance Confidence scale, and the Nottingham Health Profile, rather than in directly measured hip strength (5). Because that trial reported functional and patient-reported outcomes rather than dynamometric muscle strength, a direct effect-size comparison with our hip-strength results is not possible. Our within-group strength gains should therefore be viewed as complementary, exploratory observations rather than as confirmation of comparable effects, particularly given the absence of a control group and the small sample. Hip extensor and abductor strength nonetheless remain clinically relevant targets, as they are critical for prosthetic ambulation and fall prevention in amputees; future controlled trials directly comparing wearable sensor-guided exercise against standard care should incorporate strength outcomes alongside functional measures to enable formal, like-for-like effect-size comparison.

The mean SUS score of 75.8 exceeds the generally accepted benchmark of 68 and is comparable to the mean score of 76.64 reported in a meta-analysis of digital health applications (15). This finding suggests that the Rebee system offers usability comparable to or exceeding other health technology applications. The SUS has been validated as a reliable tool for assessing rehabilitation technology usability (17), and scores above 70 are associated with acceptable usability for widespread implementation. The consistent endorsement that home exercise programs should be part of standard therapy indicates strong patient support for integrating such technology into routine clinical care.

The variable exercise compliance (range 20%–100%, mean 68%) reflects the real-world challenges of home-based rehabilitation programs. The mean adherence observed here is broadly consistent with the moderate and highly variable adherence reported across home-based and unsupervised telerehabilitation programmes, where engagement commonly declines over time and is influenced by technological familiarity, perceived benefit, motivation, and the availability of caregiver and technical support (6, 7). In our cohort, the lower-adherence participants tended to be older or less technologically familiar and to encounter connectivity difficulties, echoing barriers described in the broader telerehabilitation literature (6). Interestingly, the weak correlation between compliance and strength improvements suggests that even moderate engagement with the exercise program may yield clinical benefits. This finding aligns with systematic review evidence suggesting that one to three exercise sessions of 20–60 min per week can improve balance, mobility, and functional outcomes in adults with LLA (4). However, the small sample size precludes definitive conclusions about dose-response relationships.

Recent systematic reviews have identified wearable technology as accounting for approximately 37% of lower limb telerehabilitation studies, with evidence supporting their utility for exercise assessment, joint angle estimation, and activity recognition (6). The Rebee system's ability to detect repetitions, measure range of motion, and enable remote monitoring addresses key requirements for effective telerehabilitation delivery. The preliminary validation of Rebee for shoulder assessment (9) provides a foundation for its application in lower limb rehabilitation, though specific validation for hip exercises in amputee populations would strengthen the evidence base.

Several technical challenges were identified that should inform future system refinements. Connectivity issues, the system requires both a WiFi internet connection and a Bluetooth link between the tablet and IMU sensor, tablet stability during exercises, and sensor sensitivity were reported concerns. These findings are consistent with broader literature on telerehabilitation implementation, which identifies technical complexity, connectivity, and user interface design as common barriers (6). Enabling offline functionality, improving system stability, and incorporating accessibility features (larger fonts, audio instructions) for users with visual impairment or lower technological familiarity would enhance usability across diverse patient populations.

The clinical implications of this study are significant for rehabilitation service delivery. Singapore's high amputation rate, combined with an aging population and increasing prevalence of diabetes, underscores the need for scalable rehabilitation solutions. Telerehabilitation using wearable sensors offers potential benefits including reduced travel burden for patients and caregivers, increased therapy accessibility, objective exercise monitoring, and efficient use of physiotherapy resources through remote supervision. The 2024 VA/DOD Clinical Practice Guidelines for Rehabilitation of Individuals with Lower Limb Amputation emphasize the importance of individualized rehabilitation approaches and support the integration of technology-enabled care where appropriate (2).

### 4.1 Limitations

Several limitations should be considered when interpreting these findings. First, the single-arm design without a control group precludes determination of whether improvements exceed those achievable with standard care alone. While participants continued receiving usual physiotherapy, the specific contribution of the Rebee intervention cannot be isolated. Second, the small sample size limits statistical power and generalizability, though it is appropriate for a pilot study. Third, the heterogeneous participant characteristics (varying amputation levels, causes, and comorbidities) introduce variability, though this also reflects real-world clinical populations. Fourth, outcome assessors were not blinded, potentially introducing measurement bias. Fifth, longer-term follow-up beyond 8 weeks would provide information on durability of strength gains and prosthetic outcomes. Additionally, recruitment was limited by several practical barriers, including prospective participants declining due to work commitments, lack of a suitable caregiver at home, reluctance to use technology-based interventions, and difficulties with learning to operate the device. These observations suggest that wearable sensor-based telerehabilitation may be most suitable for a selected subgroup of patients, such as those with adequate caregiver support, basic technological literacy, and sufficient motivation, rather than as a universal replacement for conventional therapy. Future implementation strategies should incorporate patient selection criteria to optimise uptake and adherence. Finally, economic evaluation was not conducted but would be valuable for informing clinical service implementation decisions.

### 4.2 Future directions

Based on the favorable findings from this pilot study, several directions for future research are recommended. A larger randomized controlled trial comparing Rebee-assisted home exercise to standard care physiotherapy would provide definitive efficacy evidence. Incorporation of prosthetic-specific exercises as participants progress in their rehabilitation would enhance the intervention's relevance. Introduction of gamified features and motivational prompts may enhance user engagement and compliance. Cost-effectiveness analysis comparing telerehabilitation to conventional outpatient therapy would inform resource allocation and service planning. Long-term follow-up examining prosthetic outcomes, mobility, and quality of life would establish the durability and functional significance of observed improvements.

## 5 Conclusions

This pilot study suggests that home-based telerehabilitation using the Rebee wearable sensor system is feasible, safe, and acceptable for patients with lower limb amputation. Statistically significant within-group improvements in hip extensor and abductor strength were observed over 8 weeks with mean exercise compliance of 68%; however, in the absence of a control group these changes cannot be attributed to the intervention. High system usability (SUS 75.8) and patient satisfaction support acceptability, although the small, single-arm sample means the findings are exploratory and cannot be generalized. The technology enables remote monitoring of exercise compliance and performance, potentially improving therapy accessibility while optimizing physiotherapy resources. Future research should include larger randomized controlled trials to confirm effectiveness and cost-effectiveness analyses to inform clinical service integration. With targeted improvements addressing connectivity, system stability, and accessibility features, wearable sensor-based telerehabilitation holds promise as a valuable adjunct to conventional amputee rehabilitation.

## Data Availability

The original contributions presented in the study are included in the article/Supplementary Material, further inquiries can be directed to the corresponding author.
